# Shifting From Opioids to Simple Analgesics for Emergency Care of Patients With Low Back Pain: A Secondary Analysis of the SHAPED Cluster Randomized Trial

**DOI:** 10.1001/jamahealthforum.2024.3008

**Published:** 2024-09-27

**Authors:** Claudia Côté-Picard, Danielle M. Coombs, Qiang Li, Chris G. Maher, Gustavo C. Machado

**Affiliations:** 1 Center for Interdisciplinary Research in Rehabilitation and Social Integration, Laval University, Québec City, Québec, Canada; 2 Institute for Musculoskeletal Health, Sydney Local Health District, New South Wales, Australia; 3 Physiotherapy Department, Royal Prince Alfred Hospital, Camperdown, New South Wales, Australia; 4 The George Institute for Global Health, UNSW Sydney, Sydney, New South Wales, Australia; 5 Sydney School of Public Health, Faculty of Medicine and Health, The University of Sydney, Sydney, New South Wales, Australia

## Abstract

This secondary analysis of a cluster randomized clinical trial examined a guideline-based care model for patients with low back pain, specifically whether physicians switched from opioids to low-risk or high-risk nonopioid pain medicines.

## Introduction

Low back pain (LBP) clinical guidelines advise against opioid use and instead recommend nonsteroidal anti-inflammatory drugs (NSAIDs) and/or paracetamol as first-line analgesics.^1^ Still, two-thirds of patients with LBP are administered opioids in emergency departments (EDs) in Australia,^2^ and approximately 40% of patients are administered opioids in EDs in the US.^3^

The SHAPED (Sydney Health Partners Emergency Department) trial implemented a guideline-based LBP care model at 4 EDs in Australia.^2^ The strategy reduced opioid use in EDs from 62.8% to 50.5% of episodes (odds ratio [OR], 0.57 [95% CI, 0.38-0.95]) without adversely affecting patient-reported outcomes (pain intensity, disability, quality of life, and care satisfaction).^2^ The original analyses did not assess if ED physicians switched from opioids to the recommended pain medicines or other risky pain medicines (eg, benzodiazepines and antiepileptics). These medicines present risks of misuse, addiction, and overdose, and evidence is insufficient to support their use for LBP.^4,5^ Some settings have demonstrated that reduction in opioid use may co-occur with increased use of risky pain medicines, especially gabapentinoids.^5,6^ We aimed to assess the effect of the SHAPED guideline-based intervention on nonopioid pain medicine use in ED management of LBP.

## Methods

We conducted a secondary analysis of a multicenter, stepped-wedge cluster randomized trial (Supplement 1; eMethods in Supplement 2).^2^ A 13-month control phase of usual care started in July 2017, and then each ED transitioned in a randomized order to the multifaceted clinician-targeted 4-week intervention.^2^ Adults with nonspecific or radicular LBP were included. SHAPED received ethical approval and waiver of informed consent to access electronic medical records from the Sydney Local Health District Human Research Ethics Committee (Royal Prince Alfred Hospital zone).^2^ The trial followed the CONSORT reporting guideline. We examined the proportion of LBP presentations receiving (1) each class of nonopioid pain medicines alone or in combination with opioids and/or nonopioids, (2) nonopioid pain medicines alone or no pain medicine used, and (3) NSAIDs/paracetamol alone or in combination.

We performed an intention-to-treat linear regression analysis for each outcome using SAS software, version 9.3 (SAS Institute) (Supplement 2). The intervention effect was estimated as ORs with 95% CIs. Adjusted analyses were performed with covariates of gender, age, diagnosis, day of presentation, mode of arrival, and triage category. The secondary analyses were conducted in February 2024. Statistical significance was determined with 2-sided testing (α < .05).

## Results

A total of 4625 LBP presentations were included (Figure); baseline characteristics were similar between groups.^2^ No evidence that ED physicians switched from opioids to other risky pain medicines was found (Table). NSAID use alone and NSAIDs with paracetamol had significant absolute increases of 1.4% (OR, 3.05 [95% CI, 1.24-7.52]) and 7.1% (OR, 2.05 [95% CI, 1.16-3.65]), respectively. Benzodiazepine use had a significant absolute decrease of 2.3% (OR, 0.42 [95% CI, 0.20-0.86]), and the proportion of LBP episodes treated solely with nonopioid pain medicines had an absolute increase of 10.4% (OR, 2.30 [95% CI, 1.48-3.58]). Adjusted analyses produced similar results.

**Figure.**
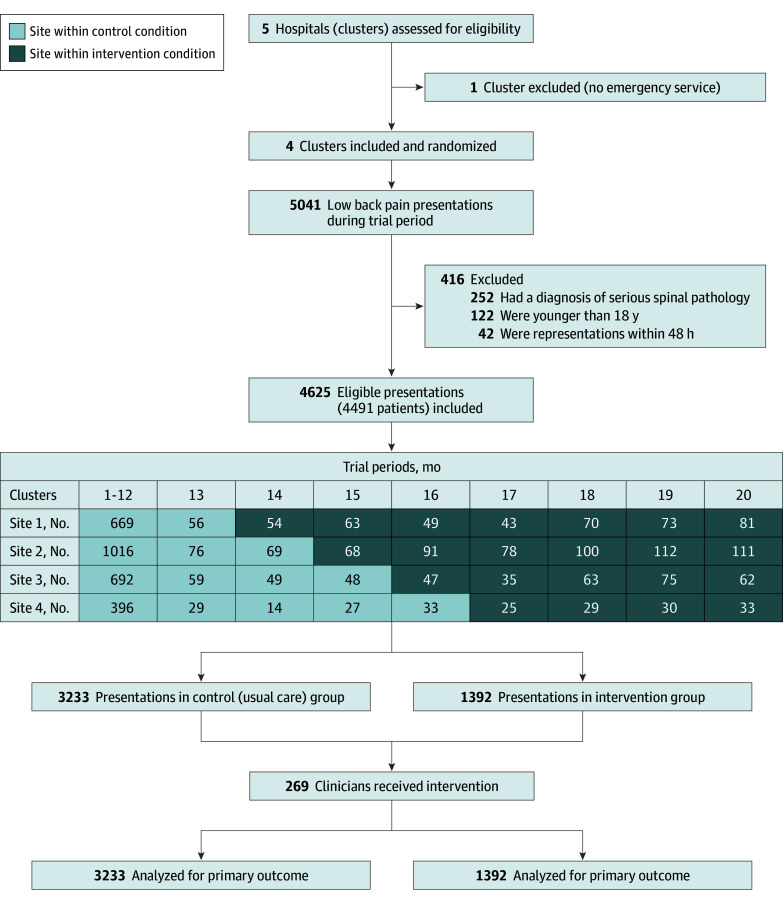
Study Flowchart This figure was adapted with permission from Coombs et al 2021, *BMJ Quality and Safety.*^2^

**Table.** Intervention Effects

Outcome	No. LBP episodes (%)		Intervention effect, OR (95% CI)	*P* value	Adjusted analysis, OR (95% CI)^a^	*P* value
	Control (n = 3233)	Intervention (n = 1392)				
Individual classes of nonopioid pain medicine alone or in combination^b^						
Benzodiazepine	249 (8.2)	81 (5.9)	0.42 (0.20-0.86)	.02	0.39 (0.18-0.83)	.01
Muscle relaxant^c^	6 (0.2)	18 (1.3)	NA	NA	NA	NA
Corticosteroid	45 (1.5)	27 (2.0)	0.62 (0.16-2.47)	.50	0.88 (0.22-3.59)	.86
Antiepileptic	170 (5.6)	88 (6.4)	2.45 (0.77-7.74)	.13	3.05 (0.96-9.69)	.06
Antidepressant	70 (2.3)	33 (2.4)	0.52 (0.13-2.12)	.36	0.67 (0.16-2.76)	.58
Paracetamol	1563 (51.6)	777 (56.4)	1.38 (0.91-2.07)	.13	1.44 (0.95-2.17)	.08
NSAID	1445 (47.7)	701 (50.9)	1.56 (1.05-2.32)	.03	1.46 (0.97-2.19)	.07
Nonopioid pain medicine only or no pain medicine^b^						
Nonopioid only	624 (20.6)	427 (31.0)	2.30 (1.48-3.58)	<.001	2.19 (1.40-3.44)	<.001
No pain medicine	500 (16.5)	254 (18.4)	0.80 (0.47-1.38)	.43	0.79 (0.45-1.37)	.39
NSAID and/or paracetamol and no other pain medicine						
Paracetamol only	143 (4.4)	77 (5.5)	1.40 (0.62-3.16)	.41	1.41 (0.63-3.17)	.41
NSAID only	132 (4.1)	77 (5.5)	3.05 (1.24-7.52)	.02	2.88 (1.16-7.14)	.02
Paracetamol plus NSAID only	295 (9.1)	225 (16.2)	2.05 (1.16-3.65)	.01	1.91 (1.06-3.44)	.03

Abbreviations: LBP, low back pain; NA, not applicable; NSAID, nonsteroidal anti-inflammatory drug; OR, odds ratio.

^a^ Adjusted analyses were performed with covariates of gender, age, diagnosis, day of presentation, mode of arrival, and triage category.

^b^ Data are missing for opioids and nonopioid medicines for control and intervention groups (n = 216 [4.8%]). Proportions for the first 2 outcomes, (1) individual classes of nonopioid pain medicine alone or in combination and (2) nonopioid pain medicine only or no pain medicine, were calculated with n = 3031 LBP episodes in the control group and n = 1378 in the intervention group.

^c^ ORs could not be calculated for muscle relaxants.

## Discussion

In addition to opioid use reduction previously reported,^2^ this secondary analysis of a cluster randomized trial showed no indication that ED physicians replaced opioid medicines with other risky pain medicines. The increase in the use of NSAIDs alone and with paracetamol indicates a shift toward nonopioid alternatives that align with guidelines for treating patients with LBP and efforts to mitigate the opioid crisis.^1^

Study limitations include uncertainty on generalizability to other settings or countries. Also, the intervention had 5 components; future studies should identify the optimal, scalable strategy to implement guidelines encouraging safer LBP case management in EDs. Nonetheless, this secondary analysis provides randomized evidence that shifting away from opioids and toward safer analgesics for patients with LBP in the ED is possible.

## Notes

Supplement 1. Trial Protocol and Statistical Analysis Plan

Supplement 2. eMethods. Supplemental Methods and Statistical Analysis eReferences

Supplement 3. Data Sharing Statement
