# Supplementary material for: Shifting From Opioids to Simple Analgesics for Emergency Care of Patients With Low Back Pain: A Secondary Analysis of the SHAPED Cluster Randomized Trial
Source: JAMA Health Forum. 2024 Sep 27;5(9):e243008. doi: 10.1001/jamahealthforum.2024.3008 (PMC11437380; doi:10.1001/jamahealthforum.2024.3008)
Supplement: Supplement 1. — Trial Protocol and Statistical Analysis Plan [file jamahealthforum-e243008-s001.pdf]

Supplement 1 - Trial protocol (published in *BMJ Open*)[1]

Introduction

Low back pain is a common presenting complaint in emergency settings. In 2015–2016 alone, there were 104 072 low back pain presentations to emergency departments in Australia, placing this condition among the top 10 reasons for emergency visits.[2] This condition is also a common reason for emergency department presentations across the globe, accounting for 4.4% of all presentations.[3] Unfortunately, many patients receive low-value care for their low back pain in the emergency department. Low-value care is broadly defined as the use of an intervention that provides patients with little to no benefits or causes harm.[4] Examples of low-value care of low back pain in emergency departments include inappropriate overuse of imaging, liberal use of opioid analgesics and unnecessary admission to hospital.

Multiple clinical guidelines exist for the management of low back pain in primary care.[5,6] Although it is unclear whether these guidelines should be applied in the emergency department, much of their recommendations may be relevant to emergency physicians and are often used to guide their practice.[7] However, the mixture of providing inappropriate care and failing to provide appropriate care in the emergency department is a clear indication that healthcare is not following clinical guidelines. For instance, about 30% of patients with non-specific low back pain receive imaging in the emergency department[8] when guidelines explicitly recommend no imaging for these cases. Imaging in the absence of suspected serious pathology does not improve patient outcomes[9] and can potentially cause harms.[10-12] Against guideline advice, around 62% of low back pain patients receive opioids in the emergency department,[13] although efficacy in pain relief has not been established for acute low back pain[14] and side effects are often serious,[15] including dependence, overdose and death. Another issue is the increasing rate of hospital admissions. More than one-third of low back pain presentations to the emergency department lead to the patient being admitted to hospital,[8] where care is likely to be similar to what could be provided in primary care.

The significant deviations from evidence-based recommendations occurring in Australian emergency departments15 make them an appropriate setting to trial an intervention based on improving care for low back pain. The Agency for Clinical Innovation (ACI) has recently launched a model of care for acute low back pain that could be applied in both primary care and emergency department settings.[16] The ACI model of care was developed in collaboration with policy-makers, clinicians, consumers and researchers and distils the high-quality evidence in this area to formulate key messages for practice (table 1). Briefly, the model provides different care pathways according to a classification based on a diagnostic triage[18] (acute or chronic non-specific low back pain, low back pain with leg pain and suspected serious spinal conditions). Risk stratification[19] is recommended to guide the amount and type of treatment provided; including personalised evidence-based health education and treatment. Lastly, follow-up reviews are scheduled to monitor individuals' progress. Passive dissemination of guidelines, such as the ACI model of care, is unlikely to change practice. We are proposing a multifaceted strategy to implement and evaluate the ACI model of care to see if this improves health service delivery and patient outcomes for low back pain at the emergency department.

Table 1. The key principles of the ACI model of care for acute low back pain

Principle 1	Assessment: history and examination
Principle 2	Risk stratification
Principle 3	Patient education
Principle 4	Active physical therapy encouraged
Principle 5	Begin with simple analgesic medicines
Principle 6	Judicious use of complex medicines
Principle 7	Cognitive behavioural approach
Principle 8	Only image those with suspected serious spinal pathology
Principle 9	Predetermined times for review

Principle 10	Timely referral and access to specialist services

Source: NSW Agency for Clinical Innovation. *Management of people with acute low back pain: model of care*. Chatswood: NSW Health, 2016. Available at: https://www.aci.health.nsw.gov.au/resources/musculoskeletal/management-of-people-with-acutelow-back-pain/albp-model.

Objectives

The overall aim of the Sydney Health Partners Emergency Department (SHaPED) trial is to implement and evaluate the ACI model of care for acute low back pain. The outcomes of the trial reflect the key messages in the model: (1) patients with non-specific low back pain do not require imaging; (2) where medicines are used, simple analgesics should be the first option; (3) patients with non-specific low back pain should be managed as outpatients.

Primary objective

The primary objective of this study is to evaluate if implementation of the ACI model of care significantly reduces the proportion of patients presenting with low back pain who receive imaging in the emergency department.

Secondary objectives

The secondary aims of the study are:

- To determine if implementation of the ACI model of care significantly reduces the proportion of patients presenting with low back pain who receive opioids in the emergency department and the proportion of patients subsequently admitted to hospital.
- To determine if implementation of the ACI model of care significantly improves patient outcomes in people who present with low back pain in the emergency department.
- To determine the cost-effectiveness of the ACI model of care compared with current emergency department practice for people who present with low back pain.
- To determine the barriers and facilitators to the implementation intervention of the ACI model of care for people who present with low back pain in the emergency department.

Methods and analysis

Study design

The Standard Protocol Items: Recommendations for Interventional Trials guidelines were followed in this report of the protocol.[20] SHaPED will use a stepped-wedge cluster randomised controlled trial design.[21] In this study design, clusters are randomised to cross from the control period (ie, unexposed to intervention) to the intervention period at regular intervals (‘steps’) until all clusters have crossed to the intervention under evaluation. This design is particularly suited to interventions aiming to improve healthcare systems as all groups eventually receive the intervention. Moreover, the process allows for comparison with control sites that have not yet implemented the intervention.

In the SHaPED trial, after a retrospective baseline observation control period of 12 months prior to randomisation, the intervention will be sequentially rolled out, with a new emergency department receiving the intervention every 4 weeks, until all participating emergency departments have received the intervention. After the implementation of the ACI model of care, the emergency departments will continue using the pathways of care outlined in the model until the end of the trial (table 2).

Table 2. SHaPED trial design

Steps (clusters)	Year 1												Year 2						
	1	2	3	4	5	6	7	8	9	10	11	12	1	2	3	4	5	6	7
ED 1																			
ED 2																			
ED 3																			
ED 4																			

Light grey: 12-month retrospective baseline control period.
Middle grey: 4-week initial intervention period.
Dark grey: sites continue with intervention plus follow-up period.
ED, emergency department; SHaPED, Sydney Health Partners Emergency Department.

Study setting

The emergency departments of one rural and three urban hospitals in New South Wales, Australia will participate in the study: Royal Prince Alfred Hospital, Concord Repatriation General Hospital, Canterbury Hospital and Dubbo Base Hospital. Investigators in the SHaPED trial are listed in Appendix 1.

Clinician participants

Clinician participants included in the SHaPED trial will be emergency clinical staff, such as physicians, nurses and physiotherapists, who routinely manage patients presenting to emergency departments with a primary complaint of low back pain. Potential clinician participants will be invited by the principal investigator of each emergency department and will receive a participant information statement. Research staff will verbally explain the information provided in this document to fully inform potential clinician participants of the risks and benefits of their participation. In addition, the research staff will be available to answer any questions to ensure that potential clinician participants fully understand the implications of their decision. A written participant consent form will be obtained from all participating clinicians prior to randomisation.

Patient participants

We will use codes from the Systematised Nomenclature of Medicine—Clinical Terms—Australian version, Emergency Department Reference Set[22] to identify low back pain presentations (Appendix 2) to the emergency departments. Presentations with codes related to low back pain with non-specific cause or those associated with neurological signs and symptoms (such as sciatica and lumbar spinal stenosis) will be included. Representations to the emergency department within 48 hours or low back pain presentations related to serious spinal pathologies (such as lumbar fracture or cauda equina syndrome) will be excluded. A random subsample of 200 patient participants from each trial period will be referred to a brief self-reported online questionnaire to evaluate the effectiveness of the implementation of the ACI model of care on patient outcomes.

Randomisation

Before the beginning of the intervention, the four hospitals will be randomly allocated the 'step' when the intervention will commence at their emergency department. Randomisation will be conducted using computer-generated random numbers by research staff. Only the research team will be aware of cluster allocation.

Intervention

A framework has been proposed to facilitate the implementation of research evidence into clinical practice, known as the knowledge-to-action process.[23] This framework links the various types of research enquiry with the key steps in the research translation cycle. The process consists of the knowledge creation cycle and the action cycle and involves end users of research (eg, policy-makers, clinicians and patients) to facilitate engagement with the implementation strategy. We will use this framework to develop a tailored intervention strategy to implement the ACI model of care at the participating emergency departments.

Implementation will begin with visits to each participating emergency department to establish collaborations and approvals. We will also assess organisational issues and potential barriers to the implementation intervention, such as intake and flow of patients with low back pain, assessment of current practices, acceptability of new model and specific roles of emergency clinicians in managing these patients. We will identify existing models of care that are used to guide management of patients presenting with low back pain at each emergency department. Then, we will work with local clinical staff to ensure that each site practices according to the full ACI model of care.

A multifaceted intervention package will be used to implement the ACI model of care at the emergency departments. Briefly, the initial 4-week intervention will consist of printed and electronic educational materials, educational seminars and educational outreach, website support, posters and an audit and feedback approach. Clinician participants will receive a copy of the model and other printed materials, including the ACI consumer information booklet, as well as access to additional online support tools outlined in the ACI model of care, such as web pages and videos, to help them educate their patients. Experienced clinicians, research staff and local opinion leaders (ie, directors of emergency medicine) will deliver the interactive educational seminars and educational outreach. An audit and feedback approach focused on the outcomes of the study will also be used to enhance our implementation programme. A detailed description of the implementation plan for the SHaPED trial can be found in Appendix 3.

The intervention will be tailored for each site by adapting knowledge resources (such as printed decision aids and patient resources) to the local context and by working with local opinion leaders to address potential barriers to implementing the ACI model of care. These instructions, measures and training materials will be hosted online during the implementation phase on The University of Sydney's website. Due to the nature of the intervention, it will not be possible to blind clinician participants to the intervention.

Sample size

Based on the effect size of 10% absolute reduction (from 30%[8] to 20%) in imaging referrals, combined with an alpha of 0.05 and assuming an intraclass correlation coefficient of 0.1, a total number of 1920 low back pain presentations (on average 480 per cluster) to emergency departments is needed for this stepped-wedge cluster trial with 80% power. A preliminary analysis revealed that there were over 2650 low back pain presentations to the participating emergency departments in 2016, showing feasibility of this trial.

Outcome measures

Clinician participants will complete a baseline questionnaire, including demographic questions. They will also be asked to indicate whether they have special interests in low back pain or musculoskeletal medicine, and if they had attended previous continuing medical education or postgraduate training on low back pain management. The outcomes to evaluate the effectiveness of the ACI model of care on health service delivery are routinely collected emergency department measures.

Primary outcome

Proportion of patients receiving any imaging (yes/ no).

Secondary outcomes

- Proportion of patients receiving advanced imaging (CT/MRI=yes, X-ray/no imaging=no).

- Proportion of patients receiving analgesic medications (topical, oral, injection). Medications will be classified according to the Anatomical Therapeutic Chemical (ATC) classification system (table 3). The ATC classification is recommended by the WHO and is widely used internationally in medication use studies:
  - Paracetamol.
  - Non-steroidal anti-inflammatory drugs.
  - Muscle relaxants.
  - Opioids.
  - Neuropathic pain medications.
  - Other.

- Proportion of patients admitted to:
  - Hospital.
  - Emergency Medical Unit.
  - Short Stay Unit.

- Time in emergency department (triage time to discharge or admission time).

- Proportion of patients referred to specialists (referral for a consultation by the emergency department):
  - Pain management.
  - Rheumatology.
  - Surgery.

- Proportion of patients representing to the emergency department within 48 hours.

- Proportion of patients readmitted to the hospital within 28 days.

- Total health system costs (including intervention costs and health service delivery costs).

Table 3. Medications per ATC classification

Group	ATC Code
Analgesics	N02B
NSAIDs	M01A; M02AA
Muscle relaxants	M03
Opioids	N02A; N01AH
Neuropathic pain medicines	N03; N06A

ATC, Anatomical Therapeutic Chemical; NSAIDs, non-steroidal anti-inflammatory drugs.

Patient outcomes will be collected using a brief online questionnaire that will measure pain intensity (Numeric Rating Scale, range 0–10). We will also use the Patient-Reported Outcomes Measurement Information System (PROMIS) to measure physical function (PROMIS Short Form—Physical Function 4a) and quality of life (PROMIS Scale—Global Health item 1) as advocated by the National Institutes of Health. We have chosen these outcomes as they are considered the three core outcome domains for clinical trials in low back pain identified in a recent Delphi study[24] and by the International Consortium for Health Outcomes Measurement.[25] Patient experience with

emergency service will be assessed using item 31 of the Emergency Department Patient Experience of Care survey advocated by the American College of Emergency Medicine.[26]

Data collection methods

In the week prior to the implementation intervention, the 12-month retrospective baseline health service delivery data will be extracted directly from participating hospitals' electronic record systems. The Sydney Local Health District (SLHD) Targeted Activity and Reporting System (STARS) will be used to access and extract data from SLHD emergency departments. STARS is data analytics programme which monitors clinician performance and service use. At Dubbo Base Hospital, health service delivery data will be extracted from its electronic record system. During the intervention, health service delivery measures will be extracted from all participating emergency departments every week until the end of the 3-month follow-up period. Data extraction will be conducted remotely for all participating emergency departments by research staff blinded to intervention allocation. Data collection through hospitals' electronic systems will also avoid additional workloads within the emergency departments.

Patient outcome measures will be collected using automated text messaging at 1 week (primary time point) and again at 2 and 4 weeks after index emergency department presentation. A random subsample of patient participants will be referred to a brief self-reported online questionnaire containing the Patient Information Statement. Completion of the online questionnaire indicates patient consent to participate in the study. Three reminder messages will be sent to non-responders, and those who do not respond to the third message will be contacted verbally via telephone.

Data will be securely stored in password-protected spreadsheets and transferred to appropriate statistical software for analysis. Spreadsheets will be regularly scrutinised for omissions and errors. Data will be archived at the Sydney School of Public Health, The University of Sydney for 15 years, after which data will be destroyed.

Statistical methods

Data analysis will be performed according to an intention-to-treat analysis, that is, clusters will be analysed according to their randomised cross-over time irrespective of whether cross-over was achieved at the desired time. First, we will investigate temporal trends in healthcare outcomes across the 12-month baseline observation period. In the situation of an underlying temporal trend, we will only include data for the previous 3 months as the baseline observation period. In our primary analysis, the 4-week implementation intervention period will be excluded, but a secondary exploratory analysis will be performed including the implementation period into the intervention group. For the primary outcome analysis, logistic regression models with a random effect for cluster, a fixed effect indicating the group assignment of each cluster at each step and a fixed effect of time (each step) will be used. Data will be analysed using SAS V.9.1.3 (SAS Institute).

Economic evaluation

An economic evaluation of the ACI model of care compared with current emergency practice will be undertaken from the health system perspective. First, we will measure the costs related to the delivery of the intervention (that is, training component, staff time and printed resources). Then, the costs related to health service delivery will be measured via data captured by the hospitals' electronic record systems. Costs will be valued based on government charges, using publicly available data. All costs will be reported in Australian dollars. Where necessary, costs will be converted to 2018 prices using the Australian Institute of Health and Welfare (AIHW) health price index. The incremental cost-effectiveness ratio (ICER) will be presented as the incremental cost per patient avoiding any imaging, opioid prescription and hospital admission.

Univariate sensitivity analyses will be conducted around key parameters likely to influence cost-effectiveness, including cost and efficacy estimates. For example, effectiveness parameters used in the economic evaluation will be

varied over the 95% CIs to assess impact on the ICER. Intervention costs, including training costs, staff time and resource costs will be collected from individual emergency departments and similarly, analysis will examine the effect on the ICER of varying these values over the range reported by participating sites. Bootstrapping will be used to estimate a distribution around costs and health outcomes and to estimate the CIs around the ICER. Results will be plotted on the cost-effectiveness plane.

Process evaluation

A process evaluation will be conducted to provide an indication of which elements of the intervention are effective and worthwhile. In the week before the implementation period and in the week after it, clinician participants will be asked to answer a questionnaire containing the Back Beliefs Questionnaire.[27] The Back Beliefs Questionnaire is a widely validated questionnaire[28] designed to measure beliefs about low back pain and will be used in our trial to assess whether the use of the ACI model of care improves beliefs about low back pain among emergency clinicians. This instrument was found to be reliable and responsive to change in a wide range of contexts, including in Australia.[29] We will also use a set of questions aimed at eliciting knowledge about the management of low back pain and attitudes of emergency clinicians towards these patients.[30] At the end of the implementation period, clinician participants will also be asked to review the content of educational materials. Potential barriers and facilitators will be investigated using qualitative interviews with clinician participants.

Ethics and dissemination

Our hypothesis is that implementation of the ACI model of care will improve health service delivery in participating emergency departments for patients presenting with low back pain: specifically decreasing the proportion of patients receiving imaging, opioids and hospital admission. If the trial results are positive, we will build on our existing strong relationships with the ACI, Sydney Health Partners and the Local Health Districts to support implementation of the ACI model of care in other emergency departments across New South Wales. As a branch of the New South Wales Ministry of Health, the ACI will be well positioned to facilitate transferability of findings. We will also disseminate the results of the trial at conferences and in scientific journals, and we will continue our successful approach of using the media to reach a lay audience and health consumers. The study resources will be made freely available on relevant websites so that jurisdictions beyond New South Wales can adopt the implementation strategy outlined in this study.

References

1. Machado GC, Richards B, Needs C, et al. Implementation of an evidence-based model of care for low back pain in emergency departments: protocol for the Sydney Health Partners Emergency Department (SHaPED) trial. BMJ Open. Apr 19 2018;8(4):e019052. doi:10.1136/bmjopen-2017-019052

2. Australian Institute of Health and Welfare. Emergency department care 2015–16: Australian hospital statistics. Canberra: Australian Institute of Health and Welfare, 2016.

3. Edwards J, Hayden J, Asbridge M, et al. Prevalence of low back pain in emergency settings: a systematic review and meta-analysis. BMC Musculoskelet Disord 2017;18:143.

4. Scott IA, Duckett SJ. In search of professional consensus in defining and reducing low-value care. Med J Aust 2015;203:179–81.

5. Qaseem A, Wilt TJ, McLean RM, et al. Noninvasive treatments for acute, subacute, and chronic low back pain: a clinical practice guideline from the american college of physicians. Ann Intern Med 2017;166:514.

6. National Institute for Health and Care Excellence. Low back pain and sciatica in over 16s: assessment and management: NICE Guideline (NG59). 2016.

7. Edlow JA. Managing nontraumatic acute back pain. Ann Emerg Med 2015;66:148–53.

8. McCaughey EJ, Li L, Georgiou A, et al. Imaging for patients presenting to an emergency department with back pain: impact on patient pathway. Emerg Med Australas 2016;28:412–8.

9. Jarvik JG, Gold LS, Comstock BA, et al. Association of early imaging for back pain with clinical outcomes in older adults. JAMA 2015;313:1143–53.

10. Webster BS, Cifuentes M. Relationship of early magnetic resonance imaging for work-related acute low back pain with disability and medical utilization outcomes. J Occup Environ Med 2010;52:900–7.

11. Sloan TJ, Walsh DA. Explanatory and diagnostic labels and perceived prognosis in chronic low back pain. Spine 2010;35:E1120–E1125.

12. Chou R, Qaseem A, Owens DK, et al. Diagnostic imaging for low back pain: advice for high-value health care from the American College of Physicians. Ann Intern Med 2011;154:181–9.

13. Friedman BW, Chilstrom M, Bijur PE, et al. Diagnostic testing and treatment of low back pain in United States emergency departments: a national perspective. Spine 2010;35:E1406–E1411.

14. Abdel Shaheed C, Maher CG, Williams KA, et al. Efficacy, tolerability, and dose-dependent effects of opioid analgesics for low back 2016;176:958–68.

15. Dowell D, Haegerich TM, Chou R. CDC guideline for prescribing opioids for chronic pain–United States, 2016. JAMA 2016;315:1624–45.

16. Machado GC, Rogan E, Maher CG. Managing non-serious low back pain in the emergency department: Time for a change? Emerg Med Australas 2017.

17. NSW Agency for Clinical Innovation. Management of people with acute low back pain: model of care. Chatswood 2016.

18. Maher C, Underwood M, Buchbinder R. Non-specific low back pain. Lancet 2017;389:736–47.

19. Hill JC, Whitehurst DG, Lewis M, et al. Comparison of stratified primary care management for low back pain with current best practice (STarT Back): a randomised controlled trial. Lancet 2011;378:1560–71.

20. Chan AW, Tetzlaff JM, Altman DG, et al. SPIRIT 2013 statement: defining standard protocol items for clinical trials. Ann Intern Med 2013;158:200–7.

21. Hemming K, Haines TP, Chilton PJ, et al. The stepped wedge cluster randomised trial: rationale, design, analysis, and reporting. BMJ 2015;350:h391.

22. Hansen DP, Kemp ML, Mills SR, et al. Developing a national emergency department data reference set based on SNOMED CT. Med J Aust 2011;194:S8–10.

23. Graham ID, Logan J, Harrison MB, et al. Lost in knowledge translation: time for a map? J Contin Educ Health Prof 2006;26:13–24.

24. Chiarotto A, Deyo RA, Terwee CB, et al. Core outcome domains for clinical trials in non-specific low back pain. Eur Spine J 2015;24:1127–42.

25. Clement RC, Welander A, Stowell C, et al. A proposed set of metrics for standardized outcome reporting in the management of low back pain. Acta Orthop 2015;86:523–33.

26. Weinick RM, Becker K, Parast L, et al. Emergency department patient experience of care survey: development and field test. Rand Health Q 2014;4:5.

27. Symonds TL, Burton AK, Tillotson KM, et al. Do attitudes and beliefs influence work loss due to low back trouble? Occup Med 1996;46:25–32.

28. Bostick GP, Schopflocher D, Gross DP. Validity evidence for the back beliefs questionnaire in the general population. Eur J Pain 2013;17:1074–81.

29. Buchbinder R, Jolley D, Wyatt M. Population based intervention to change back pain beliefs and disability: three part evaluation. BMJ 2001;322:1516–20.

30. Buchbinder R, Staples M, Jolley D. Doctors with a special interest in back pain have poorer knowledge about how to treat back pain. Spine 2009;34:1218–26.

Appendix 1. The SHaPED trial investigators

Writing Committee and Principal Investigators

Gustavo Machado, Bethan Richards, Chris Needs, Rachelle Buchbinder, Ian Harris, Kirsten Howard, Kirsten McCaffery, Laurent Billot, James Edwards, Eileen Rogan, Rochelle Facer, David Lord Cowell, Chris Maher.

Participating sites and Local Investigators

Royal Prince Alfred Hospital: Matthew Oliver, Danielle Coombs, Ruth Perrot.

Canterbury Hospital: Matthew Chu, Mona Marabani.

Concord Repatriation General Hospital: Daniel Harrison, Leslie Barnsley.

Dubbo Base Hospital: Kristy Hatswell.

Data Monitoring Committee

Sydney Local Health District: Mauricio Oliveira, Noel Baidya, Hannah Storey, Rachael Knoblanche.

Collaborators

Westmead Hospital: Matthew Vukasovic, Nicholas Manolios, Katherine Maka.

Royal North Shore Hospital: Rob Day, Rodger Laurent.

NSW Agency for Clinical Innovation: Matthew Jennings, Robyn Speerin.

Sydney Health Partners: Nobby Alcala.

Macquarie University: Niamh Moloney.

The University of Sydney: Manuela Ferreira, Paulo Ferreira, Chris Lin

Appendix 2. SNOMED CT-AU (EDRS) codes related to low back pain presentations

DESCRIPTION	CODES
**Low back pain with non-specific cause**	
Acute low back pain (finding)	278862001
Back pain complicating pregnancy (disorder)	91957002
Backache (finding)	161891005
Blunt injury to back (disorder)	424270008
Chronic back pain (finding)	134407002
Chronic low back pain (finding)	278860009
Coccyx sprain (disorder)	209571002
Complaining of low back pain (finding)	161894002
Degeneration of lumbar intervertebral disc (disorder)	26538006
Displacement of lumbar intervertebral disc without myelopathy (disorder)	20021007
Exacerbation of backache (finding)	135860001
Low back pain (finding)	279039007
Low back strain (disorder)	300956001
Lower back injury (disorder)	282766005
Lumbar spondylosis (disorder)	239880009
Lumbar sprain (disorder)	209565008
Mechanical low back pain (finding)	279040009
Pain in the coccyx (finding)	34789001
Sacral back pain (finding)	61486003
Spasm of back muscles (finding)	203095000
Sprain of ligament of lumbosacral joint (disorder)	209548004
Stiff back (finding)	249921008
Strain of back muscle (disorder)	262965006
Strain of tendon of back (disorder)	262975009
**Low back pain with neurological signs and symptoms**	
Acute back pain with sciatica (finding)	247366003
Acute sciatica (disorder)	307176005
Chronic sciatica (disorder)	307177001
Injury of lumbar nerve roots (disorder)	24300005
Injury of sciatic nerve (disorder)	86269002
Lumbago with sciatica (finding)	202794004
Lumbago-sciatica due to displacement of lumbar intervertebral disc (disorder)	46960006
Lumbar disc prolapse with radiculopathy (disorder)	202735001
Lumbar radiculopathy (disorder)	128196005
Sciatica (disorder)	23056005
Spinal stenosis of lumbar region (disorder)	18347007
**Low back pain due to serious pathology**	
Abscess of back (disorder)	309083007
Abscess of back, except buttock (disorder)	19284003
Cauda equina syndrome (disorder)	192970008
Closed fracture lumbar vertebra (disorder)	207957008
Collapse of lumbar vertebra (disorder)	308758008
Compression fracture of lumbar spine (disorder)	426646004
Concussion and edema of lumbar spinal cord (disorder)	212360005
Contusion of back (disorder)	11437003
Contusion of lower back (disorder)	284062002
Crush fracture of lumbar vertebra (disorder)	281933002
Disc prolapse with myelopathy (disorder)	202728009
Discitis (disorder)	2304001
Fracture of coccyx (disorder)	125871005

Fracture of lumbar spine (disorder)	125608002
Fracture of lumbar spine and/or pelvis (disorder)	207986006
Injury of cauda equina (disorder)	230614002
Lumbar disc prolapse with myelopathy (disorder)	202731005
Multiple fractures of lumbar spine and/or pelvis (disorder)	207993005
Open dislocation of coccyx (disorder)	44237008
Open fracture of lumbar vertebra with spinal cord injury (disorder)	48956000
Open fracture of sacrum AND/OR coccyx with spinal cord injury (disorder)	65491009
Traumatic dislocation of joint of lumbar vertebra (disorder)	129166009
Traumatic dislocation of lumbosacral joint (disorder)	129161004

SNOMED CT-AU (EDRS), Systematized Nomenclature of Medicine – Clinical Terms – Australian Version (Emergency Department Reference Set)

Appendix 3. SHaPED Implementation strategy and intervention description

The implementation plan for the Sydney Health Partners Emergency Department (SHaPED) trial has been adapted from: Jabbour M, Reid S, Polihronis C, Cloutier P, Gardner W, Kennedy A, Gray C, Zemek R, Pajer K, Barrowman N, Cappelli M. Improving mental health care transitions for children and youth: a protocol to implement and evaluate an emergency department clinical pathway. Implement Sci. 2016;11:90.

1. Create implementation team:

a) Obtain support from clinical leads and administration heads at the four emergency departments. Formalise a partnership agreement between institutions.

b) Recruit and engage study champions at each emergency department. Team members to include: emergency physicians, physiotherapists, nurses, managers, and clinical educators.

c) Develop a working group and form a steering committee at each emergency department to provide oversight on implementation progress.

d) Establish meeting schedule: local steering committee to meet twice a week and report to study supervisors every week during the implementation period.

2. Assessment:

a) Review and discuss the existing models of care for low back pain at the four emergency departments and recommend adaptation to facilitate adoption of the new model.

b) Conduct an environmental assessment and identify typical pathway of care for a patient presenting with low back pain at each emergency department.

c) Identify practices and processes that require development or change in order to support the implementation strategy.

d) Identify internal and external stakeholders who will be impacted by the new model and therefore require education and support to implement it.

3. Plan strategy for change:

a) Identify leadership support required for implementation phase.

b) Identify and engage influential clinical champions who will effectively drive change.

c) Revise or develop policies as needed.

d) Develop a knowledge translation strategy to support practice change, such as shared staff meetings, educational rounds, peer-to-peer mentoring.

e) Identify factors that will support practice change, such as engaging all potential stakeholders, scheduling champions and clinicians to enable attendance at meetings and face-to-face education sessions, facilitating the development of relationships between emergency physicians and other clinical staff, conducting audits or monitor specific data indicators that will support practice change.

f) Identify factors that may create a barrier for practice change in the emergency department, including attitudes and beliefs about low back pain management, and lack of clinician expertise/comfort to treat this population.

g) Develop strategies to manage barriers, such as communication, education, opportunities to develop relationships within and between clinicians and service provider.

4. Implementation:

a) Provide clinician information package:

• Deliver printed copies of the ACI Model of care (full version and executive summary) to clinician participants.

• Create a list of "red flags" to screen for serious pathologies from the ACI Model of care and deliver a printed version to clinician participants.

• Create posters outlining the '10 principles' of the ACI model of care, as well as the clinical pathways and place them at key locations of each participating emergency department.

• Inform clinician participants about and provide them access to online videos and other printed (such as the ACI consumer information booklet) and electronic educational materials to educate patients with low back pain at emergency discharge.

b) Provide patient information package:

• Encourage clinician participants to provide a printed copy of the ACI consumer information booklet to patients with low back pain during emergency department visit.

• Where most of the patient population do not speak English, encourage clinician participants to provide a copy of the Emergency Care Institute (ECI) Patient Factsheet for low back pain (available in six languages).

• Create posters outlining four myths of low back pain management and placed them at the reception area of each emergency department

c) Deliver clinician education:

• Educational seminars will be delivered by an experienced clinician (Dr Chris Needs) at week 1 of the intervention period. Booster sessions in the first week will also be conducted by local investigators (such as Directors of Emergency Medicine, clinical educators) as required, as well as in weeks 2 to 4.

• The educational seminars will be conducted primarily during the existing regular clinical staff meetings, but additional sessions will be scheduled to reach all emergency clinicians. The format of the seminars consists of a mini-lecture and interactive group discussions and will last for 40 to 60 minutes.

• During the educational seminars, clinician participants will be trained on history taking and examination of patients with low back pain, on how to use SNOMED diagnosis codes, and will be encouraged to follow the recommendations in the ACI model of care to manage these patients, with focus on the key outcomes of this study (that is, imaging, opioids, and inpatient admission rates).

• During weeks 1 to 4, individual meetings with clinician participants will be scheduled as required to cover the key messages and principles outlined in the ACI model of care. There will be at least one educational outreach visit to each clinician in weeks 1 to 4 and they can request additional if they have any concerns. Clinician participants can also seek advice from clinical educators by email.

d) Develop audit and feedback focussed on study outcomes

• Each emergency department and clinician participant will receive at the first educational seminar session an emergency department level feedback on the 12-month retrospective data performance against the outcomes of this study (that is, imaging, opioids, inpatient admission rates).

- This audit and feedback approach will be repeated each month after the implementation of the model of care during the regular emergency staff meetings until the end of the follow-up period.

- Clinician participants at the Sydney Local Health District (SLHD) will be encouraged to use the SLHD Targeted Activity and Reporting System (STARS) to monitor the emergency department performance during and after the implementation period

Sydney Health Partners Emergency Department (SHaPED) trial

STATISTICAL ANALYSIS PLAN

22 May 2019

Trial registration

Australia New Zealand Clinical Trials Registry: ACTRN 12617001160325

Main author
A/Professor Laurent Billot
The George Institute for Global Health
University of New South Wales
T: +61-2-8052-4581
lbillot@georgeinstitute.org

Table of Contents

1 Study design 4
1.1 Overview 4
1.2 Objectives 4
1.3 Outcomes 5
1.3.1 Primary outcome 6
1.3.2 Secondary outcomes 7
1.3.3 Sample size 11
2 Statistical analysis 11
2.1 Analysis principles 11
2.2 Data set analysed 12
2.3 Trial profile 13
2.4 Baseline comparisons 13
2.5 Patients and clinicians' characteristics and baseline comparisons 13
2.6 Analysis of the primary outcome 14
2.6.1 Main analysis 14
2.6.2 Treatment of missing data 15
2.6.3 Subgroup analyses 15
2.7 Sensitivity analyses of the primary outcome 15
2.7.1 Adjusted analyses 15
2.7.2 Analysis including the intervention period 16
2.8 Analysis of secondary outcomes 16
2.8.1 Binary outcomes 16
2.8.2 Continuous outcomes 16
2.8.3 Patient reported outcomes 16
3 References 17

1 Study design

1.1 Overview

The Sydney Health Partners Emergency Department (SHaPED) trial used a stepped wedge cluster randomised controlled trial design to evaluate an implementation strategy of the Agency for Clinical Innovation (ACI) model of care for acute low back pain in emergency departments (ED). In this study design, clusters are randomised to cross from the 'control period' (i.e. unexposed to intervention) to the 'intervention period' at regular intervals (steps) until all clusters have crossed to the intervention under evaluation.

In the SHaPED trial, after a retrospective baseline period of 12 months prior to randomisation, the implementation strategy was sequentially rolled out, with a new ED receiving the implementation strategy every 4 weeks, until all EDs implemented the model of care. Then, all EDs were followed-up and monitored for three months. For the primary analysis, the 'intervention period' was defined as the post-implementation and follow-up periods. The 'control period' is defined as the 12-month retrospective and prospective baseline control periods. Table 1 below illustrates the SHaPED trial design.

Table 1. Sydney Health Partners Emergency Department (SHaPED) trial design

Steps (clusters)	2017						2018												2019	
	Jul	Aug	Sep	Oct	Nov	Dec	Jan	Feb	Mar	Apr	May	Jun	Jul	Aug	Sep	Oct	Nov	Dec	Jan	Feb
ED 1														x						
ED 2															x					
ED 3																x				
ED 4																	x			

12-month retrospective baseline period
Prospective baseline control period
x 4-week implementation period
Post-implementation period
3-month follow-up period

1.2 Objectives

The overall aim of the SHaPED trial was to evaluate a multi-faceted strategy to implement the ACI model of care for acute low back pain in people who present with low back pain in the ED. The outcomes of the trial reflect the key messages in the model of care:

(1) patients with non-specific low back pain do not require imaging;
(2) where medicines are used, simple analgesics should be the first option;
(3) patients with non-specific low back pain should be managed as outpatients.

Primary objective

The primary objective of this study is to evaluate if implementation of the ACI model of care significantly reduces the proportion of patients presenting with low back pain who receive lumbar imaging in the ED.

Secondary objectives

The secondary objectives of the study are:

- To determine if implementation of the ACI model of care significantly reduces the proportion of patients presenting with low back pain who receive opioids in the ED and the proportion of patients admitted to hospital from ED.
- To determine if patient outcomes are not worse with implementation of the ACI model of care in people who present with low back pain in the ED.
- To determine the cost-effectiveness of the ACI model of care compared with usual ED practice for people who present with low back pain.
- To determine the barriers and facilitators to the implementation intervention of the ACI model of care for people who present with low back pain in the ED.

1.3 Outcomes

Health service use outcomes were collected for all eligible low back pain presentations between August 2017 and February 2019 directly from participating hospital's electronic medical records. Eligible low back pain presentations included adults >18 years with a discharge diagnosis related to low back pain (with or without radicular symptoms) in the ED identified using selected codes from the Systematised Nomenclature of Medicine Clinical Terms - Australian version, Emergency Department Reference Set (SNOMED CT-AU EDRS) (Table 2). Presentations with a SNOMED CT-AU EDRS diagnosis code related to serious spinal pathologies, such as vertebral fracture, infection or malignancy, were excluded. Re-presentations within 48 hours to the same ED during the study period were also excluded.

Table 2. SNOMED CT-AU codes related to low back pain presentations

Low back pain with non-specific cause	**Codes**
Acute low back pain (finding)	278862001
Back pain complicating pregnancy (disorder)	91957002
Backache (finding)	161891005
Blunt injury to back (disorder)	424270008
Chronic back pain (finding)	134407002
Chronic low back pain (finding)	278860009
Coccyx sprain (disorder)	209571002
Complaining of low back pain (finding)	161894002
Degeneration of lumbar intervertebral disc (disorder)	26538006

Displacement of lumbar intervertebral disc without myelopathy (disorder)	20021007
Exacerbation of backache (finding)	135860001
Low back pain (finding)	279039007
Low back strain (disorder)	300956001
Lower back injury (disorder)	282766005
Lumbar spondylosis (disorder)	239880009
Lumbar sprain (disorder)	209565008
Mechanical low back pain (finding)	279040009
Pain in the coccyx (finding)	34789001
Sacral back pain (finding)	61486003
Spasm of back muscles (finding)	203095000
Sprain of ligament of lumbosacral joint (disorder)	209548004
Stiff back (finding)	249921008
Strain of back muscle (disorder)	262965006
Strain of tendon of back (disorder)	262975009
**Low back pain with radicular symptoms**	
Acute back pain with sciatica (finding)	247366003
Acute sciatica (disorder)	307176005
Chronic sciatica (disorder)	307177001
Injury of lumbar nerve roots (disorder)	24300005
Injury of sciatic nerve (disorder)	86269002
Lumbago with sciatica (finding)	202794004
Lumbago-sciatica due to displacement of lumbar intervertebral disc (disorder)	46960006
Lumbar disc prolapse with radiculopathy (disorder)	202735001
Lumbar radiculopathy (disorder)	128196005
Sciatica (disorder)	23056005
Spinal stenosis of lumbar region (disorder)	18347007

Patient-reported outcomes were collected using automated text messaging at one week (primary time point) and again at two and four weeks after index ED presentation. Between July 2018 and December 2018, all eligible patients presenting to the participating EDs were invited to complete a brief self-reported online survey at ED discharge. Reminder messages were sent to non-responders, and those who did not respond to the reminder message were contacted via telephone. We invited a total of 807 eligible patients and 416 (52%) agreed to participate and completed week-1 survey. Of those, 360 (87%) completed week-2 and 351 (84%) week-4 surveys.

1.3.1 Primary outcome

- Proportion of patients receiving any lumbar imaging (yes/no). Any lumbar imaging will include the following radiology procedures:
  - XR Chest and Spine 2 Regions

- XR Chest and Spine 3 Regions
- XR Coccyx
- XR Lumbar Sacrum
- XR Pelvis
- XR Pelvis and Hip Left
- XR Pelvis and Hip Right
- XR Pelvis Hip and Femur Left
- XR Pelvis Hip and Femur Right
- XR Sacroiliac Joints
- XR Sacrum
- XR Skeletal Survey
- XR Spine 4 Region
- XR Spine Lumbar
- XR Spine Lumbosacral
- XR Spine Scoliosis Study
- XR Spine Thoracic Lumbar
- CT Brain and Spine Thoracic and Lumbar
- CT Pelvis
- CT Pelvis and Hip Left
- CT Pelvis and Hip Right
- CT Pelvis and Hips Bilateral
- CT Pelvis and Spine
- CT Sacrum
- CT Spine 2 regions
- CT Spine Lumbar
- CT Spine Lumbar and Pelvis
- CT Spine Cervical Thoracic Lumbar
- CT Spine Thoracic Lumbar
- CT Spine Whole
- MRI Pelvis
- MRI Sacrum
- MRI Spinal Cord Compression
- MRI Spine 2 Regions (User defines)
- MRI Spine Lumbar
- MRI Spine Whole

1.3.2 Secondary outcomes

- Proportion of patients receiving advanced imaging (CT/MRI=yes, X-ray/no imaging=no)

- Proportion of patients receiving any strong opioid medication. Any strong opioid will include the following medications:
    - Buprenorphine
    - Fentanyl
    - Hydromorphone
    - Morphine
    - Oxycodone
    - Pethidine
    - Tapentadol
- Proportion of patients receiving any opioid medication. Any opioid will include the following medications:
    - Alfentanil
    - Buprenorphine
    - Codeine phosphate
    - Codeine-aspirin
    - Codeine-ibuprofen
    - Codeine-paracetamol
    - Codeine-paracetamol-doxylamine
    - Dextropropoxyphene
    - Dextropropoxyphene-paracetamol
    - Dihydrocodeine
    - Fentanyl
    - Hydromorphone
    - Morphine
    - Nicomorphine
    - Oxycodone
    - Oxycodone-naloxone
    - Pethidine
    - Remifentanil
    - Sufentanil
    - Tapentadol
    - Tramadol
    - Tramadol-paracetamol
- Proportion of patients receiving other analgesic medications (topical, oral, injection). Other medications were classified according to the Anatomical Therapeutic Chemical (ATC) classification system (table 3). The ATC classification is recommended by the WHO and is widely used internationally in medication use studies:
    - Paracetamol (N02BE01)
    - Non-steroidal anti-inflammatory drugs (M01A, M02AA):
        - Aspirin

    - Celecoxib
    - Diclofenac
    - Diclofenac topical
    - Etoricoxib
    - Ibuprofen
    - Ibuprofen topical
    - Ibuprofen-paracetamol
    - Indomethacin
    - Ketoprofen
    - Ketorolac
    - Mefenamic acid
    - Naproxen
    - Parecoxib
    - Piroxicam
    - Piroxicam topical
    - Sulindac
- Muscle relaxants (M03):
    - Baclofen
    - Dantrolene
    - Orphenadrine
    - Orphenadrine-paracetamol
    - Vecuronium
- Corticosteroids (H02):
    - Cortisone
    - Dexamethasone
    - Hydrocortisone
    - Methylprednisolone
    - Prednisolone
    - Prednisone
    - Triamcinolone
- Benzodiazepine derivates (N05BA):
    - Alprazolam
    - Bromazepam
    - Clobazam
    - Clonazepam
    - Diazepam
    - Flunitrazepam
    - Lorazepam
    - Midazolam
    - Nitrazepam
    - Oxazepam
    - Temazepam
    - Zolpidem

        - Zopiclone
    - Antiepileptics (N03A):
        - Carbamazepine
        - Ethosuximide
        - Gabapentin
        - Lacosamide
        - Lamotrigine
        - Levetiracetam
        - Oxcarbazepine
        - Phenobarbital
        - Phenytoin
        - Pregabalin
        - Sodium valproate or valproic acid
        - Tiagabine
        - Topiramate
        - Vigabatrin
        - Zonisamide
    - Antidepressants (N06A):
        - Amitriptyline
        - Bupropion
        - Clomipramine
        - Dosulepin
        - Doxepin
        - Duloxetine
        - Duloxetine
        - Fluoxetine
        - Imipramine
        - Mianserin
        - Mirtazapine
        - Moclobemide
        - Nortriptyline
        - Paroxetine
        - Reboxetine
        - Sertraline
        - Venlafaxine
        - Vortioxetine
- Proportion of patients admitted from ED to:
    - Hospital ward
    - Emergency Medical Unit (EMU) or Short Stay Unit (EDSSU)
- Time in ED (triage time to discharge or admission time)
- Proportion of patients referred for a consultation in the ED:
    - Pain management

  - Rheumatology
  - Surgery

- Proportion of patients representing to the same ED within 48 hours
- Total health system costs (including intervention costs and health service delivery costs)
- Pain intensity (Numeric Rating Scale, range 0–10)
- Physical function (PROMIS Short Form—Physical Function 4a)
- Quality of life (PROMIS Scale—Global Health item 1)
- Satisfaction (EDPEC survey item 31)

1.3.3 Sample size

Based on the effect size of 10% absolute reduction (from 30% to 20%) in lumbar imaging referrals (primary outcome), combined with an alpha of 0.05 and assuming an intraclass correlation coefficient of 0.1, a total number of 1,920 low back pain presentations (on average 480 per cluster) to EDs is needed for this stepped-wedge cluster trial with 80% power. For patient-reported outcomes, we have run a power calculation for pain intensity (range 0–10). We have assumed a non-inferiority margin of 1-point (i.e. we accept a mean difference in pain scores of up to 1 unit). We also assumed a standard deviation of 2.5 and an ICC of 0.05 based on previous low back pain trials. The sample size required for 80% power is 30 subjects per ED, per period/month. Given that the 'core trial' contains five periods, this means a total of 150 subjects per ED or 600 overall.

2 Statistical analysis

2.1 Analysis principles

- Data analysis will be performed according to an intention-to-treat analysis, that is, clusters will be analysed according to their randomised cross-over time irrespective of whether cross-over was achieved at the desired time.
- All tests are to be two-sided with a nominal level of α set at 5%.
- Subgroup analyses (see Section 2.5.3) will be carried out irrespective of whether there is a significant treatment effect on the primary outcome. These analyses will also be unadjusted.
- No formal adjustments for multiplicity will be applied. However, the outcomes will be categorised by the degree of importance (primary *vs* secondary), and only a limited number of subgroup analyses are pre-specified. The results will be interpreted in this context.
- Analyses will be conducted primarily using SAS software (version 9.3 or above).

2.2 Data set analysed

The implementation intervention periods occurred on the following dates:

1. Concord Repatriation General Hospital (ED 1): 30/07/18 to 24/08/18
2. Royal Prince Alfred Hospital (ED 2): 27/08/18 to 21/09/18
3. Canterbury Hospital (ED 3): 24/09/18 to 19/10/18
4. Dubbo Base Hospital (ED 4): 22/10/18 to 16/11/18

Due to the cluster design, the group allocation (control or intervention) for a patient will be determined by the site and by the period during which they participated. All patients will be regardless of the interventions actually received, thus adhering to the ITT analysis principle. The different analyses periods are defined in Table 3. A patient will be counted in a period if the day of his/her ED presentation falls into that period.

Table 3. Definition of analysis period for each emergency department (ED)

Periods	Dates	ED 1	ED 2	ED 3	ED 4	Periods
1	Jul 2017	xxx	xxx	xxx	xxx	12-month retrospective baseline
2	Aug 2017	xxx	xxx	xxx	xxx	
3	Sep 2017	xxx	xxx	xxx	xxx	
4	Oct 2017	xxx	xxx	xxx	xxx	
5	Nov 2017	xxx	xxx	xxx	xxx	
6	Dec 2017	xxx	xxx	xxx	xxx	
7	Jan 2018	xxx	xxx	xxx	xxx	
8	Feb 2018	xxx	xxx	xxx	xxx	
9	Mar 2018	xxx	xxx	xxx	xxx	
10	Apr 2018	xxx	xxx	xxx	xxx	
11	May 2018	xxx	xxx	xxx	xxx	3-month baseline
12	Jun 2018	xxx	xxx	xxx	xxx	
13	01/07/18 to 29/07/18	xxx	xxx	xxx	xxx	Core stepped-wedge trial
14	30/07/18 to 25/08/18	xxx	xxx	xxx	xxx	
15	26/08/18 to 22/09/18	xxx	xxx	xxx	xxx	
16	23/09/18 to 20/10/18	xxx	xxx	xxx	xxx	
17	21/10/18 to 16/11/18	xxx	xxx	xxx	xxx	
18	17/11/18 to 16/12/18	xxx	xxx	xxx	xxx	3-month follow-up
19	17/12/18 to 16/01/19	xxx	xxx	xxx	xxx	
20	17/01/19 to 16/02/19	xxx	xxx	xxx	xxx	

Pre-implementation (control)
During implementation (not analysed)
Post-implementation (intervention)

2.3 Trial profile

The flow of patients through the study will be displayed in a CONSORT diagram.

2.4 Baseline comparisons

Description of the baseline characteristics will be presented for each of the four EDs. This will be done by aggregating data collected over the 12-month retrospective baseline period. Discrete variables will be summarised by frequencies and percentages. Percentages will be calculated according to the number of clusters with available data. Continuous variables will be summarised by using mean and standard deviation (SD), and median and interquartile range (IQR, Q1-Q3).

In addition, we will investigate temporal trends in health service use outcomes across the 12-month retrospective baseline control period for each ED. This will be done by summarising each primary and secondary outcome for each month within each ED and displaying the estimates (proportion or mean) together with their 95% confidence intervals on a graph. Should a visual inspection of the longitudinal plots suggest an underlying temporal trend, we will only include data for the previous three months (periods 11 to 13) as the baseline period to be used for the main model. The visual assessment of temporal trends over the baseline period will be completed by fitting a regression (logistic or linear) to assess the significance of time (month) slopes. The linear model will include a fixed term for time (months) as a continuous variable and a random effect of ED to account for clustering.

2.5 Patients and clinicians' characteristics and baseline comparisons

Description of the baseline characteristics will be presented by treatment period (i.e. pre-intervention or post-intervention). Discrete variables will be summarised by frequencies and percentages. Percentages will be calculated according to the number of patients in whom data are available. Continuous variables will be summarised by using mean and SD, and median and IQR (Q1-Q3). No adjustment for clustering will be applied when summarising baseline characteristics.

Baseline measures for all eligible low back pain presentations will be tabulated with at least the following variables outlined below:

- Age
- Gender
- Socioeconomic status
- ED Diagnosis
- Day of ED presentation

- ED presentation hour
- ED mode of arrival
- ED triage category
- Length of ED stay
- Proportion of presentations admitted to a hospital ward
- Length of stay in hospital ward

Characteristics of clinician participants will be tabulated with at least the following variables outlined below:

- Gender
- Age
- Main profession
- Country of professional training
- Clinical experience in main profession (years)
- Clinical experience in emergency medicine (years)
- Average number of low back pain patients seen per week

2.6 Analysis of the primary outcome

2.6.1 Main analysis

The primary endpoint is a binary variable indicating whether a patient received any lumbar imaging (yes/no). The primary analysis will be conducted using a logistic regression with a random effect for cluster (hospital ED), a random effect for clinician nested within cluster, a fixed effect indicating the group assignment of each cluster at each step and a fixed effect of time (each step). The model will be specified as follows:

$$\mathrm{logit}\ [\ P(Y_{ijk}=1)\ ] = \alpha + \beta\ Group_{ij} + \delta_j + u_i + v_{ijk} + e_{ijk} \qquad (1)$$

where:

Y_{ijk}	denotes the outcome of subject k $(k=1,\ldots,m_{ij})$ in period j $(j=1,2)$ within ED i $(i=1,\ldots,\ N)$ where m_{ij} indicates the number of subjects in period j within ED i
α	is the intercept
$Group_{ij}$	is a dummy variable indicating group allocation during period j for cluster i with $Group_{ij}$ = 1 post-intervention, and $Group_{ij}$ = 0 pre-intervention
β	is the parameter of interest estimating the treatment effect and where e^{β} is the odds ratio (OR)
δ_j	is a fixed period effect
u_i	is a random cluster effect with $u_i \sim N(0,\sigma_u^2)$

v_{ijk} is a random clinician effect which is nested within cluster with $v_{ijk} \sim N(0, \sigma_v^2)$

e_{ijk} are individual-level errors following a binomial distribution

The effect of the intervention will be presented as the OR of receiving any lumbar imaging and its 95% confidence interval (CI). In case of convergence issues caused by the inclusion of two random effects, we will remove the random clinician effect and keep the random cluster effect.

2.6.2 Treatment of missing data

Given the ED dataset only records lumbar images that occurred, we will assume that a missing data point indicates that the corresponding procedure did not occur. The primary outcome (i.e. lumbar imaging) will therefore not contain any missing data. However, manual data extraction from patient's medical records were conducted for some secondary outcomes (i.e. opioid medication), since some EDs did not have electronic medication system (eMEDS) available. No imputation of secondary or patient reported outcomes will be performed.

2.6.3 Subgroup analyses

The following subgroup analyses will be carried out for the primary outcome only:

- Age (<65 *versus* ≥65 years)
- Socioeconomic status (measured by patient's postcode - SEIFA)
- Australasian Triage Scale (ATS) category (ATS 1 to 5)
- SNOMED diagnosis codes (non-specific low back pain *versus* radicular low back pain)
- ED mode of arrival

The analysis for each subgroup analysis will be performed by adding the subgroup variable as well as its interaction with the intervention as fixed effects to the logistic regression model used for the primary analysis (see Section 2.6.1). Within each subgroup, summary measures will include raw counts and percentages within each treatment arm, as well as the OR for treatment effect with 95% CI. The results will be displayed on a forest plot including the P value for heterogeneity corresponding to the interaction term between the intervention and the subgroup variable.

2.7 Sensitivity analyses of the primary outcome

2.7.1 Adjusted analyses

The logistic regression model described in Section 2.6.1 will be re-run after adjustment for the following covariates: gender, age (continuous), ED diagnosis, day of ED presentation, ED mode of arrival and ATS category.

2.7.2 Analysis including the intervention period

A secondary exploratory analysis will be performed including the implementation period into the intervention group (see definition in Section 1.1). This analysis will be performed both unadjusted and adjusted; however, no subgroup analysis will be applied.

2.8 Analysis of secondary outcomes

2.8.1 Binary outcomes

All binary secondary outcomes will be analysed using the same strategy as the one applied to the primary outcome, that is using both an unadjusted and adjusted logistic regression model and both by excluding and including the intervention period, thus giving four models. Subgroup analyses will not be conducted on secondary outcomes.

2.8.2 Continuous outcomes

Continuous outcomes such as time in ED, will be analysed using the same strategy as the binary outcomes; however, logistic regression will be replaced with linear regression (normal distribution and an identity link function) and the effect of the intervention will be estimated as the modelled mean difference and its 95% CI.

2.8.3 Patient reported outcomes

All patient reported outcomes will be treated as continuous outcomes and therefore analysed following the strategy described in Section 2.8.2.

3 References

1. Machado GC, Richards B, Needs C for the SHaPED trial Investigators, et al Implementation of an evidence-based model of care for low back pain in emergency departments: protocol for the Sydney Health Partners Emergency Department (SHaPED) trial BMJ Open 2018;8:e019052.
2. Hussey MA, Hughes JP. Design and analysis of stepped wedge cluster randomized trials. Contemp Clin Trials 2007;28:182–91.
