# Supplementary material for: Shifting From Opioids to Simple Analgesics for Emergency Care of Patients With Low Back Pain: A Secondary Analysis of the SHAPED Cluster Randomized Trial
Source: JAMA Health Forum. 2024 Sep 27;5(9):e243008. doi: 10.1001/jamahealthforum.2024.3008 (PMC11437380; doi:10.1001/jamahealthforum.2024.3008)
Supplement: Supplement 2. — eMethods. Supplemental Methods and Statistical Analysis eReferences [file jamahealthforum-e243008-s002.pdf]

Supplemental Online Content

Côté-Picard C, Coombs DM, Li Q, Maher CG, Machado GC. Shifting from opioids to simple analgesics for emergency care of patients with low back pain: a secondary analysis of the SHAPED cluster randomized trial. *JAMA Health Forum.* 2024;5(9):e243008. doi:10.1001/jamahealthforum.2024.3008

eMethods. Supplemental Methods and Statistical Analysis
eReferences

This supplemental material has been provided by the authors to give readers additional information about their work.

© 2024 Côté-Picard C et al. *JAMA Health Forum.*

eMethods

Design and participants

The present report and the previous one[1] followed the Consolidated Standards of Reporting Trials (extension for stepped-wedge, cluster-randomised trials).[2] The complete protocol has been published elsewhere.[3] Hospitals from the Sydney Local Health District and an affiliated rural hospital in NSW, Australia, were eligible if they had an ED and used Cerner PowerChart electronic medical record system. Data were retrospectively collected in electronic medical records from July 2017 to June 2018 and prospectively from July 1st to 31st, 2018, for the control phase of usual care. Then, each ED transitioned in a randomised order to the intervention and were followed-up for at least 3 months. The intervention period went from August to November 2018 and the follow-up period ended in February 2019.

Physicians, nurses and physiotherapists who managed patients with LBP in the ED were recruited. Presentations of patients 18 years and older with non-specific or radicular LBP were eligible for inclusion in the analysis. Re-presentations within 48 hours and presentations with a diagnosis of serious spinal condition were excluded. The investigators, clinicians and patients could not be blinded to the intervention, but the outcome measures were extracted independently from the research team by hospital data managers. The SHaPED trial received ethical approval and waiver of consent to access electronic medical records from the Sydney Local Health District (Royal Prince Alfred Hospital zone) Human Research Ethics Committee (protocol number X17-0043).[1]

Intervention

The SHaPED trial used a multifaceted clinician-targeted 4-week intervention to implement a guideline-based model of care for LBP in EDs. It included five components: (i) educational seminars from experienced rheumatologists and physiotherapists, (ii) educational materials, (iii) provision of non-opioid pain management strategies, (iv) education on fast-track referral to outpatient services, and (v) audit and feedback.[1] A key message was that when a pain medicine was used, the first option should be a simple analgesic (NSAID and/or paracetamol).[1]

© 2024 Côté-Picard C et al. *JAMA Health Forum.*

Statistical analysis

The SHaPED statistical analysis plan was published in Open Science Framework Preprints and the analyses of the present study are based on it[4]. Analyses were conducted in SAS v9.3. We performed an intention-to-treat linear regression analysis for each outcome, with a random effect for cluster (ED) and clinician nested within cluster, a fixed effect for the group assignment of each cluster at each step and a fixed effect of time (each step). The intervention effect was estimated as odds ratio (OR) and its 95% CI. Adjusted analyses were performed with covariates of gender, age, diagnosis, day of presentation, mode of arrival, and triage category. The tests were two-sided with an alpha level set at 0.05.

eReferences

1. Coombs DM, Machado GC, Richards B, et al. Effectiveness of a multifaceted intervention to improve emergency department care of low back pain: a stepped-wedge, cluster-randomised trial. BMJ Qual Saf. Oct 2021;30(10):825-835. doi:10.1136/bmjqs-2020-012337

2. Hemming K, Taljaard M, McKenzie JE, et al. Reporting of stepped wedge cluster randomised trials: extension of the CONSORT 2010 statement with explanation and elaboration. BMJ. Nov 9 2018;363:k1614. doi:10.1136/bmj.k1614

3. Machado GC, Richards B, Needs C, et al. Implementation of an evidence-based model of care for low back pain in emergency departments: protocol for the Sydney Health Partners Emergency Department (SHaPED) trial. BMJ Open. Apr 19 2018;8(4):e019052. doi:10.1136/bmjopen-2017-019052

4. Billot L, Coombs D, Machado GC. Sydney Health Partners Emergency Departments (SHaPED) trial - Statistical Analysis Plan. OSF Preprints. 2019:1-23. doi:10.31219/osf.io/9g6cx

© 2024 Côté-Picard C et al. *JAMA Health Forum.*
