# Supplementary material for: Shifting From Opioids to Simple Analgesics for Emergency Care of Patients With Low Back Pain: A Secondary Analysis of the SHAPED Cluster Randomized Trial
Source: JAMA Health Forum. 2024 Sep 27;5(9):e243008. doi: 10.1001/jamahealthforum.2024.3008 (PMC11437380; doi:10.1001/jamahealthforum.2024.3008)
Supplement: Supplement 3. — Data Sharing Statement [file jamahealthforum-e243008-s003.pdf]

Data Sharing Statement

Côté-Picard. Shifting From Opioids to Simple Analgesics for Emergency Care of Patients With Low Back Pain. *JAMA Health Forum*. Published September 27, 2024.
doi:10.1001/jamahealthforum.2024.3008

Data
Data available: No

Additional Information
Explanation for why data not available: Due to information governance restrictions imposed by organisations governing data access, we are unable to share the trial data unless applicants secure the relevant permissions.
